# Simple Acupoints Prescription Flow Chart Based on Meridian Theory: A Retrospective Study in 102 Dogs

**DOI:** 10.1155/2013/129315

**Published:** 2013-05-20

**Authors:** Jong-Ho Jeong, Joo-Young Song, Hyo-Gwon Jo, Ji-Min Kim, Samuel-S. Yoon, Chul Park, Seunghyun Kim, Seong-Soo Roh, Bong Hyo Lee, Chae Ha Yang, Hee Young Kim

**Affiliations:** ^1^Boo-Young Animal Hospital, Gigeum-dong, Namyangju-si, Gyeonggi-do 472-080, Republic of Korea; ^2^Sarang Animal Hospital, Guro-3-dong, Gurogu, Seoul 152-053, Republic of Korea; ^3^Jakjeon 24 Hour Animal Hospital, Jakjeon 1-dong, Gyeyang-gu, Incheonsi, Gyeonggi-do 407-060, Republic of Korea; ^4^Jun Animal Hospital, Galsan-dong, Dongangu, Anyangsi, Gyeonggi-do 431-088, Republic of Korea; ^5^Twin City Animal Hospital, 869 South Street, Fitchburg, MA 01420, USA; ^6^Department of Veterinary Internal Medicine, College of Veterinary Medicine, Chonbuk National University, Jeonju-si, Jeollabuk-do 561-756, Republic of Korea; ^7^College of Korean Medicine, Daegu Haany University, Daegu 706-828, Republic of Korea; ^8^Department of Physiology, College of Korean Medicine, Daegu Haany University, Daegu 706-828, Republic of Korea

## Abstract

To help the clinicians prescribe acupoints easily and effectively, we developed one simple flow chart to select acupoints. This study aimed to evaluate the usefulness of flow chart to select acupoints in dogs. Total 102 dogs showing intervertebral disc disease (IVDD) (*n* = 12), vomiting (*n* = 11), diarrhea (*n* = 2), abdominal pain (*n* = 5), cough (*n* = 66), or epilepsy (*n* = 6) received acupuncture treatment according to the chart, and its outcomes were evaluated as regards clinical symptoms, duration, treatment numbers, and recovery time. Dogs (8/8) with IVDD from grades I to III recovered over periods of 5 days to 6 weeks after 1–12 treatments, while 1/4 dogs with grade IV recovered over 7 weeks after 15 treatments. Vomiting dogs with acute/subacute (*n* = 8) and chronic symptoms (*n* = 3) required about 1 and 7 treatments to recover fully, respectively. All dogs (*n* = 5) with abdominal pain showed fast relief within 24 hours after acupuncture. Two diarrhea cases recovered over 2–9 days after 1-2 treatments. Fifty-four of 66 coughing dogs were recovered by 1-2 treatments. And 5 of 6 epilepsy dogs under a regular acupuncture treatment had no epileptic episode during followup of 12 months. These results suggest that this flow chart can help the clinicians prescribe acupoints effectively.

## 1. Introduction

Acupuncture had been used in human and veterinary practice for thousands years in Eastern Asia including China (called traditional Chinese medicine), Republic of Korea (called Korean medicine), Japan (called Kampo medicine), and other Asian countries until it was banned in their counties to promote Western medicine in the early 1900s. Since the visit of President Nixon to China in 1972 and articles about surgery in conscious patients under acupuncture anesthesia first hit the West, there has been an explosion of interest about acupuncture in the United States, Europe, and other countries. Veterinary acupuncture has also been resurrected and developed rapidly during the past 30 years. Veterinary acupuncture organizations have been established in most developed countries including those of North and South America, Europe, the Middle East, Australia and New Zealand, South Africa, Republic of Korea, and many Asian countries. Also, some veterinary schools have included class on acupuncture in their curriculum [1].

The number of certified veterinary acupuncturists and veterinarians wishing to study acupuncture has increased remarkably in the last decade. Now, many veterinarians and veterinary students spend much effort and expense to learn traditional oriental medical (OM) theory in an intensive course. They aim at integrating acupuncture into their practice but they discover quickly that OM theories are extremely complex and confusing to be applied in practice. Therefore, novices in the art science of veterinary acupuncture tend to rely strongly on “Cookbook Acupuncture,” in which a routine set of acupoints is used to treat certain diseases. Though “Cookbook Acupuncture” is very useful for beginners, it is not specific for individual cases and its results are not as good as those of acupuncture adapted by experts for each specific case. Therefore, many practitioners using “Cookbook Acupuncture” become frustrated when they achieve outstanding results in some cases but no response in others. 

To apply acupuncture most effectively, practitioners must make an OM diagnosis for each case, by using OM theory. That theory includes *Yin-Yang*, *Zang*-*Fu* organs, Channel (Meridian) Theory, and point indications. According to the OM diagnosis, two sets of acupoints are chosen: (a) main (essential) points and (b) helper (supporting) points. This combination of main and helper points is called an acupoints prescription or combination for each individual case. The correct choice of points plays a key role in acupuncture's success. When appropriated on combination, the main and helper acupoints produce synergic effects that boost the clinical efficacy of acupuncture. In contrast, some acupoints may counter the beneficial effects of the main acupoints through opposing actions [2–4]. For clinicians to select effective acupoints for individual cases efficiently, it is essential that they understand OM diagnosis, the properties and interactions of the Channels, the functions of each acupoint, and combination methods of their acupoints. However, it is difficult, especially for new acupuncturists, to comprehend OM theories completely, memorize all the information about acupoints, and build each treatment plan according to individual patient's condition. Therefore, we sought to develop for clinicians one simple acupuncture flow chart based on OM theories and diagnosis. Over a 5-year period, this flow chart has been modified through its clinical application. We now introduce one simple acupoints prescription chart and its clinical cases. 

## 2. Materials and Methods

### 2.1. Acupoints Prescription Chart (Figure 1)

The proposed chart was designed to select acupoints in a total of 5 steps. 

In Step 1, according to the location of disease in the body (viz interior or exterior), two or three acupuncture points of 8 Extraordinary Channel points and 6 Command points were selected (Figures 2(a) and 2(b)).

In Step 2, one of 8 Influential points was chosen according to the body components (*Zang*, *Fu*, *Muscle*, *Bone*, *Qi*, *Blood*, *Vessel*, and *Marrow*) affected by disease (Figure 3).

In Step 3, the Back-*Shu* or Abdomen-*Mu* points sensitive to palpation were selected. To find the sensitive *Shu* (Figure 4(a)) or *Mu* (Figure 4(b)) points on back and abdomen, the right hand palpated the skin on Back-*Shu* and Abdomen-*Mu* points while the left hand was positioned on abdomen. The points at which dogs exhibited the protective abdominal reflex, skin twisting, growling, grunting, and head-turning toward the palpated point during palpation were considered as sensitive Back-*Shu* or Abdomen-*Mu* points, which were diagnosed as the affected internal organs [5] for the next Step 4. Those points were used as treatment points.

In Step 4, according to the diagnostic results from Step 3 or patients' main symptoms, the associated Channels were chosen (Figures 5(a) and 5(b)), and two main points, *Yuan *(source) point and *He *(sea) point, on the Channels were used for acupuncture. Some points on the Channel frequently were chosen according to the patient's condition, such as acute/subacute (<7 days), chronic (>7 days), emergency, or joint pain.

Lastly, in Step 5, local points or empirical points for each case were added. 

### 2.2. Cases (*n*  =  102)

The 102 dogs that received acupuncture treatment by 5 clinicians according to simple acupuncture flow chart were reviewed.  Table 1 shows case signalment including age, sex, and disease duration. Ages of dogs varied from 3 months to 15 years (average age, 4.12 years). Forty-six percent (47/102) were males, and 54% (55/102) were females. Purebred dogs accounted for 52% (53/102) and were represented by 9 pure breeds (11 miniature Poodles, 14 Maltese, 6 Shih Tzus, 2 Chihuahuas, 1 Siberian husky, 8 Yorkshire terriers, 2 English cockers, 5 Pugs, and 4 miniature Schnauzers). The remaining 48% (49/102) were mixed-breed dogs. Routine diagnostic tests including fecal examination, urinalysis, blood tests, and/or radiography were performed in private practice to determine the possible cause.

Acupuncture was prohibited in cases with evidence of foreign body on radiography or infectious viral disease (distemper or parvovirus) by commercial ELISA kits. All except epilepsy cases received acupuncture treatment only without conventional Western medicine. Cases were classified into 6 groups, according to main symptoms: intervertebral disc disease (IVDD) (*n* = 12), vomiting (*n* = 11), diarrhea (*n* = 2), abdominal pain (*n* = 5), cough (*n* = 66), and epilepsy (*n* = 6) (Table 1). IVDD was further classified as grade I to IV: grade I = no neurologic signs except back pain, grade II = conscious proprioceptive deficit and ambulatory paraparesis, grade III = nonambulatory paraparesis, and grade IV = non-ambulatory paraparesis with loss of deep pain perception [6]. Gastrointestinal disorders including vomiting, diarrhea, and abdominal pain were classified by the duration to clinical presentation as acute (<2 days), subacute (2–7 days), or chronic (>7 days). Application of acupuncture for cough was restricted to subacute cases (duration, 2–7 days), since acute cough was often a self-limiting problem that may resolve without any symptomatic/supportive therapy, or chronic cases had a risk of severe bacterial infection without antibiotics, subsequently death. In idiopathic epilepsy, one dog was 3 months at onset of epileptic seizure and the remaining 5 dogs were over 1 year, at a frequency of 2–4/month under control of phenobarbital and potassium bromide therapy. Five of the 6 dogs showed generalized and symmetrical seizure and one was seen to have unilateral focal motor activity of the head which spread to unilateral limbs.

### 2.3. Acupuncture Treatment

**Figure 1:**
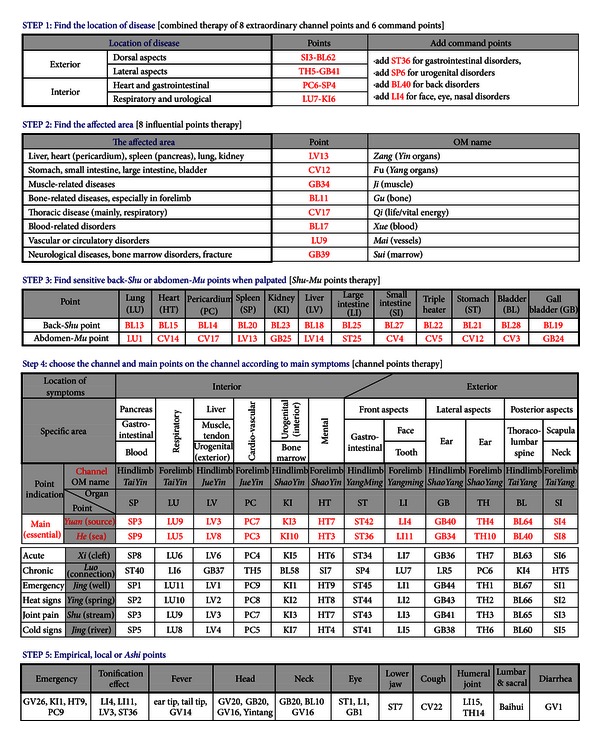
Acupoints prescription flow chart.

Acupuncture was performed by 5 veterinarians in their own practice. After selecting acupoints according to the flow chart (Figure 1), acupuncture needles (stainless steel, 0.24–0.30 mm in diameter, 15–40 mm in length) were inserted, as described in text [5] and left for 15–20 minutes with/without manipulation. Acupuncture was applied 2-3 times/week. Clinical followup was determined either by a phone call to the owners at the time of this study or by return of the dog to the veterinary hospital. Treatment was discontinued if the owner stated the symptoms had ceased and if main symptoms were apparently disappeared on laboratory or physical examination.

## 3. Results

### 3.1. IVDD (*n*  =  12)

The age of the dogs varied from 2 to 9 years with an average age of 3.6 years. The affected breeds were miniature Poodle (*n* = 3), Yorkshire terrier (*n* = 2), miniature Schnauzer (*n* = 2), Shih Tzu (*n* = 1), and mixed-breed (*n* = 4). Spinal palpation pain (hyperesthesia), when assessed by manual compression along the thoracolumbar spine, was detected at one or more levels of T11-T12 (*n* = 2), T12-T13 (*n* = 6), T13-L1 (*n* = 5), L1-L2 (*n* = 4), L2-L3 (*n* = 1), and L3-L4 (*n* = 1). In cases with severe symptoms (grade III or IV), diffuse back pain over two spinal levels was frequently observed. On plain radiographic examination, narrowed intervertebral disk space (*n* = 3), osteophyte formation (*n* = 1), or mineralized intervertebral disk (*n* = 2) was found in grade III or IV cases. According to the flow chart (Figure 1), the following points were chosen for acupuncture: SI3, BL62, and BL40 (in Step 1), GB34 (in Step 2), Back-*Shu* points at levels showing hyperesthesia on the spinal palpation (in Step 3), and BL40 and BL60 (in Step 4). Acupuncture was performed 2-3 times a week. Cases that returned to normal or cases with non-ambulatory paraparesis (grade III or IV) that improved to be able to walk and void urine and feces without assistance were considered to be recovered. All dogs (8/8, 100%) from grades I to III recovered over periods of 5 days to 6 weeks after 1–12 treatments, while only 1/4 dogs (25%), diagnosed as grade IV, recovered over 7 weeks after 15 treatments.

### 3.2. Vomiting (*n*  =  11)

The vomiting cases, diagnosed as unknown causes from diagnostic tests or not responsive to initial managements of food withdrawals and antiemetics for 1–3 days, were subjected to this acupuncture treatment. They were young (*n* = 1, 6 months) or young adult dogs (*n* = 10, average age = 3.2 yrs) and showed commonly vomiting and inappetance. The acupoints were chosen based on the flow chart: PC6, SP4, and ST36 (in Step 1), LV13 and CV12 (in Step 2), sensitive Back-*Shu*, and Abdomen-*Mu* points to palpations (in Step 3), ST36, ST42, SP3, and SP9 (in Step 4). Acute vomiting cases (<2 days; *n* = 6) were well responsive to this acupuncture treatment. Interestingly, 3 of them began to eat food within 1 hr after withdrawals of acupuncture needles and did not show any vomiting. In subacute cases (2–7 days; *n* = 2) that showed vomiting 3–5 times/day and were not responsive to initial treatment of food withdrawals (first 24 hr) and metoclopramide, one or two vomitings were noted up to 12 hr after first acupuncture and thereafter no more vomiting episodes. Chronic cases (>7 days; *n* = 3) suffered from sporadic vomiting of 2-3 times/day, frequently after food intake, recovered over 18.67 days after 6.7 times treatments and required longer periods and more treatments than acute and subacute cases.

### 3.3. Diarrhea (*n*  =  2)

A 1.5-month-old female Poodle dog (acute case) was presented with acute watery diarrhea of 2 episodes within 12 hr. Since the patient still kept normal appetite without dehydration and pyrexia and owner was most favorable toward alternative medicine use, acupuncture was first tried without routine laboratory examinations or Western medicine. The following acupoints were used: PC6, SP4, and ST36 (in Step 1), LV13, and CV12 (in Step 2), sensitive Back-*Shu* and Abdomen-*Mu* points to palpations (in Step 3), ST42, ST36, SP3, and SP9 (in Step 4), and GV1 (in the last Step 5). Increase of stools consistency and decrease of stools frequencies were observed within 48 hr after first acupuncture treatment. She returned to normal over 9 days after two acupuncture treatment. A 6-month-old male mixed dog (subacute case) was presented with acute onset (<2 days) of mild diarrhea, poor appetite, and frequent vomiting. When presented, a complete blood count and routine serum chemistry levels were within normal limits and fecal examinations including parvovirus, distemper virusو and parasites were also normal. The patient was first treated with loperamide and amoxicillin for 5 days, but the diarrhea persisted. Acupuncture was then applied at the same points as those of acute diarrhea case. He began to recover appetite on the day of acupuncture treatment and returned to normal consistent feces 2 days after acupuncture treatment.

### 3.4. Abdominal Pain (*n*  =  5)

The cases (2 Maltese, 1 Pug, 1 Cocker spaniel, and 1 miniature Poodle) were initially presented with acute abdominal discomfort, increasing abdominal pain, inappetance, and/or bloating, and they all were lesser than 2 days at duration. Physical examination was normal, except abdominal pain. There was no radiographic evidence for foreign bodies. Acupuncture was performed at the acupoints selected according to the flow chart. They all returned to normal within 24 hours after one acupuncture treatment. Interestingly, when food was offered 30 min after withdrawal of acupuncture needles, 4/5 cases began to eat immediately and did not show any abdominal pain on palpation.

### 3.5. Cough (*n*  =  66)

The coughing cases in this study consisted of 60 shelter and 6 hospital cases. On August and September 2007 in Republic of Korea, workers in two shelters noticed abrupt outbreaks of coughing dogs after rainy spell in summer, although the medications including antibiotics were done under regular shelter program. The most affected dogs were small pure or mixed breeds. Although the exact age of the shelter dogs was usually unknown, ages were estimated to be between 6 months and 7 years old, based on dentition and hair coat. The common clinical signs were cough and nasal discharge for 3–7 days. Under shelter's approval, treatment was performed using acupuncture without Western medicine, at the following acupoints: LU7, KI6, and LI4 (in Step 1), CV17 or LV13 (in Step 2), sensitive Back-*Shu* and Abdomen-*Mu* points to palpations (in Step 3), LU9 and LU5 (in Step 4), and CV22 (in Step 5). Two shelter veterinarians observed daily spontaneous coughing and nasal discharge in acupuncture-treated dogs. At 7 days after one acupuncture treatment, it was noted that 48 dogs showed no symptoms of cough and nasal discharge, and the other 12 had still cough or nasal discharge and thereafter were prescribed antibiotics. The above favorable effects were also observed in 6 hospital cases (1 Poodle, 1 Yorkshire terrier, 3 Shih Tzus, and 1 mixed-breed) within 2–7 days at onset duration of coughing. They received acupuncture treatments two times a week at the same points as those of shelter dogs. Decrease of coughing frequencies was observed on 3 days after first acupuncture and they all did not show any coughing on the next visits (on 7 days after acupuncture treatment). 

### 3.6. Epilepsy (*n*  =  6)

The 6 cases (2 Pugs, 1 Shih Tzu, 1 Maltese, 1 miniature Poodle, and 1 mixed breed) showed recurrent seizure at a frequency of 2–4/month under control of antiepileptic drugs. They were diagnosed as presumed idiopathic epilepsy based on physical and neurological examinations and hematological and serum biochemical analyses. One Maltese dog of 3 months at onset of epileptic seizure received only acupuncture treatment once a week without medication, since his owner was reluctant to give antiepileptic drugs. The other 5 dogs over 1 year at onset of epileptic seizure received regular acupuncture treatment once a month with anticonvulsants. Acupoints were chosen, as shown in (Table 2). Since acupuncture treatment, 5 of the 6 dogs (5/6, 83%) had no epileptic episode during followup of 12 months. However, a 5-year-old male Pug, presented with frequent generalized and symmetrical epilepsy during 1.5 years, did not show any changes in frequencies of epilepsy after acupuncture treatment. 

## 4. Discussion

### 4.1. The Present Acupuncture Flow Chart Follows Basic OM Principles for Point Combinations

Although various methods can be used to select effective acupoints in OM, good prescriptions must satisfy at least basic OM principles. These include (1) bilateral acupuncture, (2) combination of forelimb and hindlimb points, (3) ventral and dorsal points, and (4) local and distal points [7]. The present chart follows all of the above principles. First, each step is bilateral acupuncture. Second, Step 1 includes a combination of points on forelimb and hindlimb. Third, Step 3 is one technique of combining *Mu*-ventral and *Shu*-dorsal points. For example, in vomiting cases, ventral point CV12 and dorsal point BL21 were selected in Step 3. Lastly, a combination of local and distal points is included in Steps 4 and 5. Thus, the present flow chart is well matched with OM principles for point combination. 

### 4.2. The Proposed Chart Lists 164 Important Acupoints and Is Designed to Select Acupoints in a Total of 5 Steps

There are approximately 360 acupoints on the 14 main channels of the body. Not all these acupoints are used commonly in veterinary and human clinics. Most acupuncturists pay great attention to the application of special acting acupoints which have special therapeutic effects. These clinically important acupoints are categorized according to their own special therapeutic properties as follows: 8 Extraordinary Channel points, 6 Command points, 8 Influential points, Back-*Shu* and Abdomen-*Mu* points, *Yuan* (source) points and *Luo* (connection) points, and 5 *Shu* (transporting) points, including *He* (sea) points, *Xi* (cleft) points, and empirical points, and local points. In the present chart, the above points were organized in order of the functional ranges of acupoints (from points with broad function to points acting locally) as follows (Figure 1).

 Step 1: 8 Extraordinary Channel points (8 points) + 6 Command points (6 points),
 Step 2: 8 Influential points (8 points),
 Step 3: Back-*Shu* points (12 points) + Abdomen-Mu points (12 points),
 Step 4: 5 *Shu* (transporting) points on 12 Channels (55 points) + *Xi* (cleft) points (12 points) + *Yuan* (source) points (12 points) + *Luo* (connection) points (12 points),
 Step 5: empirical or local points (27 points).

In Step 1, Extraordinary Channel points were combined with Command points because both acupoints groups have the widest range of actions among the acupoints and have similar indications in respects of the body areas, when divided the body into exterior (lateral or back aspects) and interior (heart/gastrointestinal or respiratory/urogenital areas) or upper (face and neck) and lower parts (back). In oriental medicine, 8 Extraordinary Channels and their key points have been considered to play most important roles in balancing body *Qi*. The medical term “8 Extraordinary Channels points” dates back to 1230s and the detailed indications and methods forming 4 pairs of acupoints (i.e., PC6-SP4) were first described in the *Yizong Jinjian *(Golden Mirror of *Medicine*) written by *Wu Qian* in 1742 [8]. Based on that book, the Extraordinary Channel points are always used in a pair for disorders of the following body areas: (1) SI3-BL62-back, spine, neck, head, eye, and brain; (2) TH5-GB41-side of body, lateral sides of the lumbar area, lateral aspect of leg, sides of body, shoulders, hip, eyes, ears, and neck; (3) PC6-SP4-heart, thorax, gastrointestinal disorders, and reproductive, and (4) LU7-KI6-respiratory, lower abdominal, and urogenital disorders [9, 10]. 

**Figure 2:**
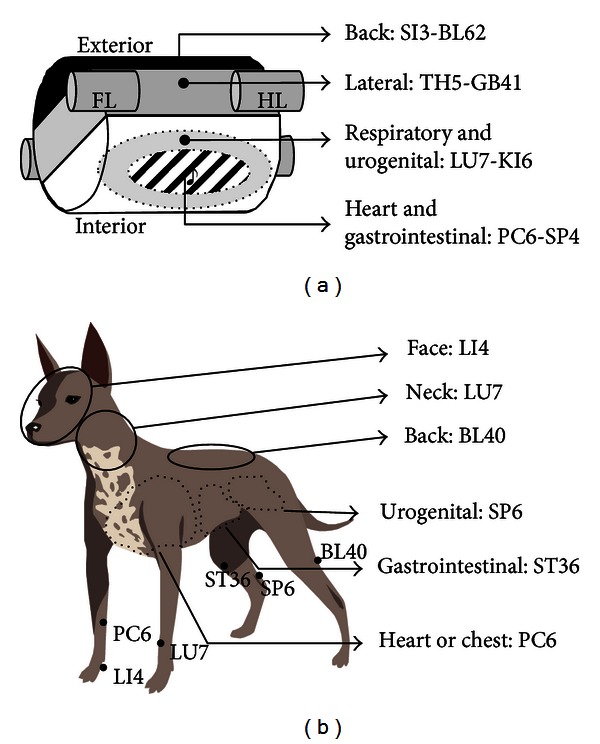
8 Extraordinary Channel points and 6 Command points (Step 1). (a) Clinical indications of 8 Extraordinary Channel points. The body was simply divided into 4 areas of exterior-back aspects, exterior-lateral aspects, interior-heart/gastrointestinal and interior-respiratory/urogenital systems, and paired acupoints (underlined) were used for disorders of their corresponding area. (b) Indication of 6 Command points. FL, forelimb; HL, hind limb.

To simplify the above theory more, we divided the body into 4 areas of exterior-back aspects, exterior-lateral aspects, interior-heart/gastrointestinal and interior-respiratory/urogenital systems, and assigned paired acupoints to each area (Figures 1 and 2(a)). In our clinical cases (Table 2), a pair of SI3-BL62 was applied to back disorders (IVDD) and bilateral epilepsy which was considered to be caused by posterior (or whole) brain lesions. A pair of TH5-GB41 was used for 1 case with unilateral epilepsy which was presumed to be due to lateral brain lesions, and LU7-KI6 was chosen for 66 coughing cases. And PC6-SP4 points were used for vomiting (*n* = 11), diarrhea (*n* = 2) and abdominal pain (*n* = 5). Previous experimental and clinical studies have successfully applied 8 Extraordinary Channel points. In one clinical study showing successful outcomes of acupuncture in IVDD dogs, paired acupoints of SI3-BL62 were used in combination with other acupoints [11]. TH5-GB41 has been used effectively for unilateral or focal headaches in human [12, 13]. LU7 and/or KI6 in combination with other acupoints has been used successfully for respiratory clinical trials [14–16]. Acupoint PC6 is extensively for treatment and prevention of vomiting [17–23] and the enhancement of cardiopulmonary functions [4, 24–26]. Wang et al. reported that acupoints at PC6 and SP4 enhance cardiac and gastrointestinal functional activities after acute myocardial ischemia through the mediation of nitric oxide (NO) [27]. 

In Step 1, theoretically, to enhance the effects of Extraordinary Channel points, 6 Command points therapy were added. In detail, the 6 Command points are 6 individual points which have been used to control diseases in 6 major body parts, abdomen (ST36), lumbar region (BL40), neck (LU7), heart (LI4), chest (PC6), and urogenital organ (SP6) [28] (Figure 2(b)). Six Command points originated from 4 Command points (ST36, LI4, LU7 and BL40), described in Chinese classic *Qiankunshengyi* (Meanings of Life between Heaven and Earth; 1402) and formed by adding two points (PC6 and SP6) later [29]. In the present study, one of 6 Command points was chosen additionally in cases of IVDD (BL40) and diarrhea/abdominal pain (ST36). Previous experimental and clinical studies support the effect of the 6 Command points which described in Step 1 of Figure 1. BL40 commonly in previous clinical trials of dogs with IVDD [11, 30]. ST36 is a acupoint, well known to be most effective for gastrointestinal disorders such as abdominal pain, diarrhea, and irritable bowel diseases [3, 31–38]. Acupuncture at SP6, a common point for urinary disorders [39, 40], has shown to decrease symptoms of urinary incontinence by stress in rats [41], diurnal symptoms associated with idiopathic bladder instability [42], and symptoms of frequency, urgency, and dysuria in female cases [43]. Although we could not conclude that the application of these combined points in Step 1 led to the present favorable outcomes, at least this flow chart allows an easy and fast approach to apply clinically the complicated OM theory concerning 8 Extraordinary Channel and 6 Command points.

**Figure 3:**
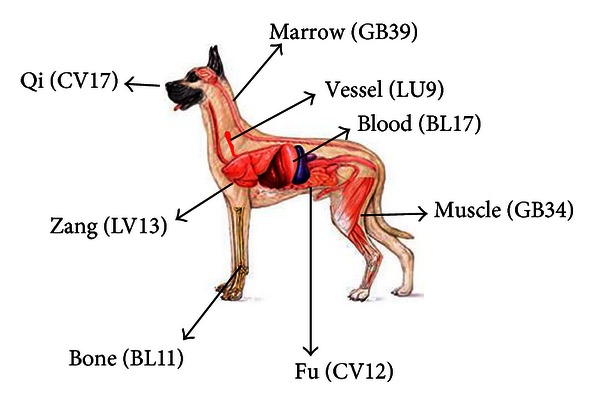
8 Influential points (Step 2). Based on OM, body consists of 8 components and each of them can be controlled by its key point (underlined).

In Step 2, one or two of 8 Influential points were selected according to the body components affected by diseases. Eight Influential points are based on a Chinese classic *Nan Jing* (*The Classic of Difficulties*) written around the 2nd century AD. It classifies the body as having 8 components: *Zang*, *Fu*, *Qi*, *Xue (Blood)*, *Ji (Muscle)*, *Mai (Vessels)*, *Gu (Bone)*, and *Sui* (*Marrow*). Each of these has a most influential point that exerts a profound effect on the function of each component [44]. Figure 3 and Step 2 in Figure 1 show 8 body components in OM, interpretation in Western medicine, and their corresponding Influential points. For example, CV12 can be used for disorders of stomach, small intestine and large intestine (*Fu *organs in OM), such as epigastric distention, abdominal pain, constipation, or diarrhea, and GB34 can be selected for muscle-related disorders (*muscle* in OM) such as muscle spasm, painful tendons, and hemiplegia. In our present studies, we chose GB34 for IVDD cases, LV13/CV12 for vomiting, diarrhea, and abdominal pain, and CV17/LV13 for cough and GB39 for epilepsy, respectively. Previous studies have included acupoint(s) of GB34 in IVDD [11, 30], LV13/CV12 in gastrointestinal disorders [34, 45, 46], CV17 in respiratory disorders [47], and GB39 in brain disorders, respectively [48].

**Figure 4:**
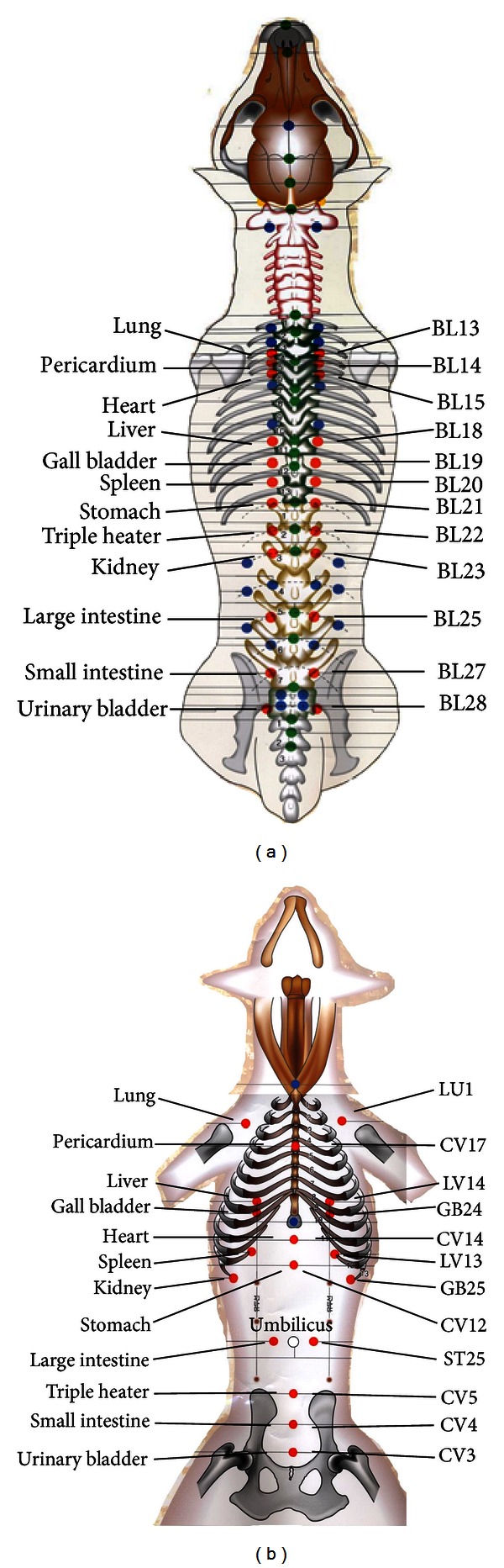
Back-*Shu* and Abdomen-*Mu* points (Step 3). (a) Back-*Shu* points (red color points) and internal organs. (b) Abdomen-*Mu* points (red color points) and internal organs.

In Step 3, Back-*Shu* and Abdomen-*Mu* points showing sensitivity to surgeon's palpation were selected for acupuncture.  Figure 4 and Step 3 in Figure 1 show the Back-*Shu* and Abdomen-*Mu* points and the associated internal organs. Oriental medicine describes that Back-*Shu* and Abdomen-*Mu* points are connected directly to the internal organs and these points often become tender, tight, or distended when the associated organs are diseased or imbalanced, and so they are used as diagnostic and treatment points [5, 49]. In support, a retrospective study of 175 dogs and cats with Back-*Shu* or Abdomen-*Mu *point sensitivity and their blood chemistry showed that there is at least a single correlation of the point sensitivity with a concurrent rise in the internal organs-associated chemistry values [49]. Back-*Shu* and Abdomen-*Mu* points are known to be related segmentally to the internal organs [50, 51]. Visceral pain is referred to segmental somatic areas. In the referred area, tender points, characterized by well-defined and localized spots and an increased sensitivity to mechanical stimuli are often found [52]. The stimulation of these tender points can in turn alleviate the visceral pain and inflammation [53–55]. In our present cases, most sensitive points were found at Back-*Shu* and Abdomen-*Mu* points around the affected internal organs and frequently some other points. In the cases with vomiting (*n* = 11) or abdominal pain (*n* = 5), sensitive points were found at BL21 (stomach *Shu* point) and/or CV12 (stomach *Mu* point) and frequently several other points such as BL17 (diaphragm point), BL18 (liver point), BL 19 (gall bladder point), BL 20 (spleen point), BL22, and BL23 (kidney point). Two diarrhea cases were sensitive at BL23/BL25 or BL21/BL24/BL25. In the cough cases, one or two sensitive points at Back-*Shu* points such as BL12 (*wind* point), BL13 (lung point), BL14 (pericardium point), or BL15 (heart point) were found. These above points were stimulated by acupuncture. 

**Figure 5:**
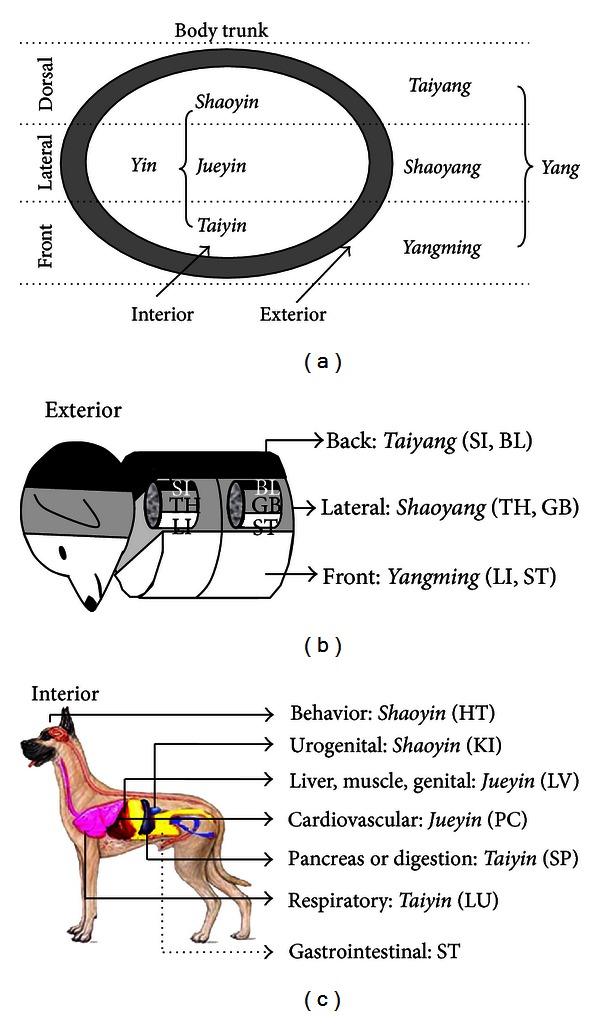
Channels and their clinical indications (Step 4). (a) Ancient anatomical terms “3 *Yang*s-3 *Yin*s” and body. *Yang* and *Yin* represent exterior and interior of body, respectively. The *Yang* (exterior of body) is divided into 3 sub-*Yang*s, namely, *Yangming* (front), *Shaoyang* (lateral), and *Taiyang* (dorsal or back). On the other hand, the *Yin* (interior of body) is divided into 3 sub-*Yin*s, namely, *Taiyin* (front organs; lung, spleen), *Jueyin* (middle organs; pericardium, liver), and *Shaoyin* (dorsal organs; heart, kidney). (b) Three *Yang* Channels and Exterior. External body is divided front, lateral and back, and their associated Channels (underlined) were used for disorders of each area. (c) Three *Yin* Channels and Interior. Each *Yin* Channel is used for treatment of their specific internal organs (a and c). Of the *Yang* Channels, the stomach (ST) Channel is especially useful to treat gastrointestinal disorders. The words in *Italic* are Chinese Pinyin. LI, large intestine Channel; ST, stomach Channel; TH, triple heater Channel; GB, gall bladder Channel; SI, small intestine Channel; BL, urinary bladder Channel; LU, lung Channel; SP, spleen Channel; PC, pericardium Channel; LV, liver Channel; HT, heart Channel; KI, kidney Channel.

In Step 4, one or two of 12 regular Channels were chosen according to main symptoms or diagnosis from Step 3, and then two Main points (*Yuan* and *He* points) were selected on the chosen Channels (Figure 1). Regular Channels are *Qi* pathways to connect the external body with internal organs (*Yin* Channels) or head (*Yang* Channels) [5]. Oriental medicine teaches that diseases occur when the *Qi *flow is disrupted in one or more Channels, which can be relieved by stimulating acupoints on the affected Channels. To differentiate the Channels which were likely to be affected by disorders, we used the “3 *Yang*-3 *Yin* theory” which has been the most important fundament for diagnosis and treatment in acupuncture medicine [56, 57] (Figure 5(a)). In ancient anatomical terms, *Yang* and *Yin* represent exterior and interior of body, respectively. In respect of treatment, *Yang* and *Yin* also represent external (exterior) and internal (interior) disorders, respectively. The *Yang* (exterior of body) is divided into 3 sub-*Yang*s, namely, *Yangming* (front), *Shaoyang* (lateral) and *Taiyang* (back or dorsal), and each of them is used for its corresponding external disorders (Figures 5(a) and 5(b) and Step 4 in Figure 1). In our present study, based on “3 *Yang*-3 *Yin* theory,” IVDD or epilepsy was diagnosed as exterior-back (*Taiyang*) disorder and thus *Taiyang* Channels (SI and BL) were chosen. Then, two Main points *Yuan* (source) and *He* (sea) points on SI and BL Channels were selected for acupuncture. *Shaoyang* Channels (TH and GB) were chosen for epilepsy with clinically presumed unilateral brain lesion (Table 2). On the other hand, the *Yin* (interior of body) is divided into 3 sub-*Yin*s, namely, *Taiyin* (front organs; lung, spleen), *Jueyin* (middle organs; pericardium, liver) and *Shaoyin* (dorsal organs; heart, kidney), and each of them is used for treatment of its specific internal organs (Figures 5(a) and 5(c)). Empirically, stomach (ST) Channel of *Yang* Channels has been used to treat internal organs (stomach and intestine), as well as exterior-front disorder. In our present study, cough was diagnosed as interior-front organ-Lung (*Taiyin* LU) disorder, and thus *Taiyin* LU Channel and its Main points (*Yuan* and *He* points) were selected for treatment. Case with vomiting, diarrhea, or abdominal pain was diagnosed as interior-front organ-spleen (*Taiyin* SP) disorder or stomach (ST) disorder, according to sensitivity at BL20 (spleen *Shu* point) or BL21 (stomach *Shu* point) and two Main points were selected on SP (spleen) or ST (stomach) Channels (Table 2). Although the above Channel theory is most important in acupuncture medicine, it is true that the theory is too difficult for general clinicians to comprehend. Therefore, we highly simplified clinical indications of each Channel and summarized it in Step 4 (Figure 1). 

In Step 5, local, empirical, or *Ashi* (local sensitive) points for each case were added. Acupuncture needling is known to produce local anti-inflammatory, analgesic, and antipyretic effects by promoting vasodilation and blood flow locally and releasing neuromodulators [58]. In the present cases, we chose GV1 in diarrhea, CV22 in cough, and GV20/GB20/GV16/*Yintang* in epilepsy, respectively. GV1 is a single acupoint in the depression ventral to the base of the tail and dorsal to the anus. It is one of the most effective acupoints to treat diarrhea in humans and animals [59–61]. Our previous studies demonstrated that acupuncture at GV1 depressed proximal colonic motility by decreasing the total duration and frequency of contractile states in conscious dogs and also had anti-inflammatory and analgesic effects in colitis rats, via endogenous opioid pathways [54, 55, 62]. CV22 has been included to treat respiratory disorders [63, 64]. GV20, GB20, GV16, or Yintang points have been used commonly for brain disorders such as headache [65]. Previous reports has shown that acupuncture at *Yintang* and/or GV20 can cause sedative effects and change bioelectrical brain activity [66]. Although we could not determine which acupoint in flow chart was most effective for the treatment of each case, each step in this flow chart provides a theoretical rationale for selection of optimal acupoints in each case, based on OM theory.

### 4.3. The Proposed Chart Can Help Clinicians Prescribe Acupoints Effectively for Various Diseases

In 1997, the NIH released a consensus statement concluding that acupuncture is effective or at least useful for the treatment of 13 conditions including low back pain, nausea and vomiting, asthma, stroke rehabilitation, headache, addiction, dental pain, menstrual cramps, tennis elbow, fibromyalgia, myofascial pain, osteoarthritis, and carpal tunnel syndrome [67]. Multiple studies have documented that acupuncture is useful in the case with low back pain or IVDD when a definitive diagnosis is made or when surgical intervention is not an option due to patient concerns such as geriatric and other underlying diseases, and acupuncture results are favorable and comparable to those of surgical treatments [30, 68–75]. In veterinary medicine, the success rates and recovery periods by acupuncture in IVDD dogs seem to vary according to the severity of disease. In clinical reports of Janssens LA [76], 90% of dogs with grade I recovered after 2-3 treatment over 1-2 week period, 90% of dogs with grade II recovered after 3-4 treatment over a 3-week period, and 80% dogs with grade III recovered after 5-6 treatments over a 6-week period. And dogs with grade IV showed poor response to acupuncture (success rate < 25%). Similarly, in our present study, 100% in dogs of grades I to III recovered over periods from 5 days to 6 weeks after 1–12 treatments, while only 1/4 dogs in grade IV recovered over 7 weeks after 15 treatments. It seemed that as the severity of IVDD increased, the recovery period and number of treatments also increased. Acupuncture treatment of idiopathic epilepsy has been documented well in the veterinary and human literature [5, 57, 68]. It was reported that acupuncture reduces seizure frequency and dosage requirements of antiepileptic drugs in epileptic dogs [77–80]. In our present study, 5/6 dogs with epileptic episode at a frequency of 2–4/month showed no seizures under combination therapy of acupuncture and anticonvulsants. It indicates that this flow chart may be useful for neurological cases with IVDD or epilepsy. 

Acupuncture is used extensively for gastrointestinal disorders, such as vomiting, diarrhea, and abdominal pain. It decreases the severity of nausea and emesis from a variety of causes in humans and dogs [18, 20, 21, 67]. In the present study, all dogs with vomiting (*n* = 11), diarrhea (*n* = 2), and abdominal pain (*n* = 5) recovered after 1–6 treatments over 1–19 days. Most acute/subacute cases (<7 days at duration) fully recovered within 1 day after one acupuncture treatment, without Western medicine. Interestingly 3/6 acute vomiting cases began to eat foods within 1 hr after withdrawal of acupuncture needles and 4/5 abdominal pain cases showed complete relief of abdominal pain within 30 min after withdrawal of acupuncture needles, with no recurrence. Similarly, in one human clinical study of 190 cases with intestinal colic pain from bacillary dysentery, simple acute appendicitis, simple acute intestinal obstruction, adhesive intestinal obstruction, or intestinal ascariasis, acupuncture at bilateral ST36 results in complete pain relief within 30 min in 85% of cases, decreased colic pain in 11%, and no response in 4% [81]. To our knowledge, although it has limitations to explain these phenomena scientifically, our results show that this flow chart can help the clinicians prescribe acupoints effectively for cases with various gastrointestinal symptoms. Acupuncture may have beneficial effects on the treatment of respiratory diseases including bronchitis and asthma in dogs and cats [82]. Although this was an uncontrolled clinical study, and not a randomised controlled clinical trial, our results that acupuncture improved 54/66 coughing cases showed that acupuncture methods using this flow chart can be used to help the treatment of respiratory cases.

## 5. Conclusion

This acupoints prescription chart is based on oriental medical (OM) theory and includes information concerning OM diagnosis, function of the Channels, *Zang*-*Fu* theory, clinically important acupoints, and combination methods of their acupoints. It has been modified through clinical trials since first edition in 2003 and used widely and successfully to various clinical cases such as neurological, respiratory, and gastrointestinal disorders in Korean veterinary clinics. We believe that this chart helps beginners or clinicians to select effective acupuncture points easily and quickly. However, more powerful methods of well-designed randomized and controlled trials are needed to confirm the efficacy of this flow chart on various diseases. 

## Figures and Tables

**Table 1:** Case signalment.

Diseases	Severity	Duration	Case (*n*)	Sex		Age (year)
				Male	Female	
IVDD	Grade I		1	1		3.00
	Grade II		1	1		2.00
	Grade III		6	2	4	2.87
	Grade IV		4	2	2	5.25
Vomiting	Acute	<2 days	6	2	4	2.77
	Subacute	2–7 days	2		2	3.50
	Chronic	>7 days	3	3		3.00
Diarrhea	Acute	<2 days	1		1	1.50
	Subacute	2–7 days	1	1		0.60
	Chronic	>7 days				
Abdominal pain	Acute	<2 days	5	2	3	2.54
	Subacute	2–7 days				
	Chronic	>7 days				
Cough	Acute	<2 days				
	Subacute	2–7 days	66	30	36	4.55
	Chronic	>7 days				
Epilepsy		<1 year	1	1		0.6
		>1 year	5	2	3	5.76

Total			102	47	55	3.75

**Table 2:** The acupoints selected according to flow chart and treatment outcome.

Diseases	Severity	Case (*n*)	The used acupoints	Treatment numbers	Recovery times (day)	Recovered animals/cases
IVDD	Grade I	1	Step 1: SI3, BL62, BL40 Step 2: GB34 Step 3: sensitive Back-*Shu* points Step 4: BL40, BL64 (BL Channel) Step 5: Baihui, *Ashi* points near lesion along BL Channel	1.0	5.00	1/1
	Grade II	1		8.0	49.00	1/1
	Grade III	6		12.5	38.67	6/6
	Grade IV	4		15.0	49.00	1/4

Vomiting	Acute Subacute Chronic	6 2 3	Step 1: PC6, SP4, ST36 Step 2: LV13, CV12 Step 3: sensitive Back-*Shu* and/or Abdomen-*Mu* points Step 4: ST36, ST42, SP3, SP9 (ST, SP Channels)	1.0 1.0 6.7	0.84 1.00 18.67	6/6 2/2 3/3

Diarrhea	Acute	1	(Same as those of vomiting) + GV1	2.0	9.00	1/1
	Subacute	1		1.0	2.00	1/1
	Chronic	0				

Abdominal pain	Acute	5	(Same as those of vomiting)	1.0	0.62	5/5

Cough	Acute Subacute Chronic	0 66 0	Step 1: LU7, KI6, LI4 Step 2: CV17 or LV13 Step 3: sensitive *Shu* and/or *Mu* points Step 4: LU5, LU9 (LU Channel) Step 5: CV22	1.9	7.02	54/66

Epilepsy	<1 year >1 year	1 5	Step 1: SI3, BL62 for posterior brain lesion or TH5, GB41 for unilateral brain lesion Step 2: GB39 Step 3: sensitive *Shu* and/or *Mu* points Step 4: SI4, SI8, BL40, BL64 for posterior brain lesion (SI, BL Channels) or TH4, TH10, GB34, GB40, for unilateral brain lesion (TH, GB Channels) Step 5: GV20, GB20, GV16, *Yintang *	1/week 1/week	— —	1/1 4/5
